# Earlier Visual N1 Latencies in Expert Video-Game Players: A Temporal Basis of Enhanced Visuospatial Performance?

**DOI:** 10.1371/journal.pone.0075231

**Published:** 2013-09-18

**Authors:** Andrew J. Latham, Lucy L. M. Patston, Christine Westermann, Ian J. Kirk, Lynette J. Tippett

**Affiliations:** 1 School of Psychology, the University of Auckland, Auckland, New Zealand; 2 Centre for Brain Research, the University of Auckland, Auckland, New Zealand; 3 Department of Biopsychology, Ruhr-University, Bochum, Germany; University of Groningen, Netherlands

## Abstract

Increasing behavioural evidence suggests that expert video game players (VGPs) show enhanced visual attention and visuospatial abilities, but what underlies these enhancements remains unclear. We administered the Poffenberger paradigm with concurrent electroencephalogram (EEG) recording to assess occipital N1 latencies and interhemispheric transfer time (IHTT) in expert VGPs. Participants comprised 15 right-handed male expert VGPs and 16 non-VGP controls matched for age, handedness, IQ and years of education. Expert VGPs began playing before age 10, had a minimum 8 years experience, and maintained playtime of at least 20 hours per week over the last 6 months. Non-VGPs had little-to-no game play experience (maximum 1.5 years). Participants responded to checkerboard stimuli presented to the left and right visual fields while 128-channel EEG was recorded. Expert VGPs responded significantly more quickly than non-VGPs. Expert VGPs also had significantly earlier occipital N1s in direct visual pathways (the hemisphere contralateral to the visual field in which the stimulus was presented). IHTT was calculated by comparing the latencies of occipital N1 components between hemispheres. No significant between-group differences in electrophysiological estimates of IHTT were found. Shorter N1 latencies may enable expert VGPs to discriminate attended visual stimuli significantly earlier than non-VGPs and contribute to faster responding in visual tasks. As successful video-game play requires precise, time pressured, bimanual motor movements in response to complex visual stimuli, which in this sample began during early childhood, these differences may reflect the experience and training involved during the development of video-game expertise, but training studies are needed to test this prediction.

## Introduction

Playing video-games has become a past-time of choice for current generations, allowing individuals to engage both socially and competitively with other players across the globe. Today’s modern action video games (e.g., *CounterStrike: Global Offensive*, *StarCraft II*, *Defense of the Ancients 2*, *Guildwars 2*) present players with complex visual environments that require flexible attentional resources. Multiple items must be simultaneously processed and identified as either relevant or irrelevant to in-game goals. Furthermore, objects in modern video games are not passive, but rather, through the integration of artificial intelligence and multiplayer capabilities, learn and adjust to the player as the game progresses. Success is contingent on the player’s ability to execute precise bimanual motor movements in response to these complex visual cues. Cumulating evidence suggests that extensive video-game play may lead to enhanced visual attention and executive control, generalising beyond the context of the video game (e.g., [1,2,3]). To date, however, few studies have assessed the underlying neural basis of the enhanced cognitive abilities of video game-players.

Beginning with the seminal findings of Green and Bavelier [1], expert video-game players (VGPs) have been shown to have superior performance on a wide-range of visuospatial and attentional tasks. These include: Flanker, Enumeration, Useful Field of View and Attentional Blink tasks [1], superior stimulus-response mapping [4], superior visual sensitivity [5], superior cross-modal sensory precision [6], reduced backwards masking [7], reduced task-switching costs [8], superior endogenous attention [9], and superior resolution for stored visual information [10]. Feng and colleagues have shown these enhancements last for at least four months after video-game play has completely ceased [11,12].

The paradigms typically utilized in these studies are computerized and require rapid target detection with manipulation of distractor difficulty and target eccentricity. Arguably such paradigms may simply measure the specific skills trained by video-games, and as a result, do not provide direct evidence that the cognitive proficiency of VGPs generalizes beyond the general training environment. A small number of studies, however, appear to demonstrate that video-game play may shape some fundamental aspects of the visual system. Green and Bavelier [13] found that expert VGPs were able to discriminate the correct orientation of significantly smaller Ts than non-VGPs during a visual crowding paradigm, suggesting they may possess superior visual acuity. Li, Polat, Makous and Bavelier [14] found that expert VGPs were significantly more accurate than non-VGPs on a standard contrast sensitivity paradigm. Finally, Buckley, Codina, Bhardwaj and Pascalis [15] found that central and peripheral visual fields measured using Goldmann kinetic perimetry were around 1000deg^2^ larger than non-VGPs.

Very few neuroimaging studies have examined the potential neural correlates of superior visuospatial and attentional performance of VGPs. In the first reported neuroimaging study, 8 non-VGPs underwent PET scanning while playing the video-game *Tetris* both before, and after, daily practice sessions of 30-45 minutes for 4 to 8 weeks and were compared with 16 control participants passively viewing visual stimuli [16]. Individuals who played video-games showed a significant decrease in whole brain glucose metabolism in the second scan, with improved *Tetris* performance inversely correlated with levels of glucose metabolism, suggesting more efficient utilization of neural circuitry.

Mishra, Zinni, Bavelier and Hillyard [17] compared expert VGPs and matched non-VGPs on a selective attention task while recording a 62-channel EEG. Participants were required to respond to numerical presentations in a cued letter stream, while ignoring distractor steams. Mishra et al. examined the steady state visual evoked potential (SSVEP), an EEG component thought to reflect the attentional demands of an attended stimuli, and found no significant group differences for attended stimuli, but significantly reduced SSVEP toward unattended stimuli in the expert VGPs. This suggests that expert VGPs may show a superior ability to suppress, or disregard, irrelevant stimuli. Expert VGPs also showed a significantly larger P300 component in response to numerical targets under a high perceptual load. Mishra and colleagues suggest that this result may show expert VGPs possess a greater sensitivity to task-relevant stimuli under high load.

Bavelier, Achtman, Mani and Föcker [18] compared the activation between expert VGPs and non-VGPs in the fronto-parietal attentional network and motion sensitive visual area, MT, on a visual search task. Participants indicated whether a diamond or square was in a ring of shapes surrounding a central fixation cross. This occurred under both low and high perceptual loads. Only non-VGPs showed a significant increase in the activation of the fronto-parietal attentional network in response to greater perceptual load. Bavelier and colleagues suggest that expert VGPs may be able to rely on automatized attentional allocation even under high perceptual load. Conversely, non-VGPs may need to switch to a more ‘online’ approach to attentional allocation in order to successfully perform the same task.

Expertise in humans can be accompanied by structural changes in the specific brain regions and pathways associated with that expertise (e.g., taxi drivers [19]; bilingual individuals [20]; musicians [21,22]). Similarly, inter-individual variation in task performance can be reflected in variation in specific related white matter pathways (e.g., mental rotation [23]; bimanual co-ordination [24]). Expert musicians, who show enhanced performance on visuospatial tasks (e.g., [25,26,27]) also have reduced asymmetry in the inter-hemispheric transfer time (IHTT) of visual information measured by visual evoked potentials, in contrast to the IHTTs of matched non-musicians [28]. Although there are clear differences in the skills involved in expert musicianship and expert video-game play, both proficiencies require the translation of complex visual cues into precise bi-manual motor movements and the adjustment of performance based on sensory feedback. In both forms of expertise, training often begins during early childhood and continues through much of adolescence, a period in which the brain is most malleable and continues to develop

The objective of the following study was to use EEG to examine components of the underlying neurophysiological basis of visuospatial and attentional performance of expert VGPs. Specifically we examined callosal functioning of expert VGPs on a task that required transfer of simple visual information as well as absolute occipital N1 latencies. We predicted that expert VGPs would have a more equal speed of transfer of visual information between the two hemispheres than non-VGPs, perhaps facilitating a greater ability to recruit the left hemisphere during tasks that require visuospatial attention. We used a standard task in which participants respond when detecting simple stimuli that are presented to each visual field individually, while a concurrent 128-channel EEG was recorded continuously. The latencies of occipital event-related potentials (ERPs) in the hemisphere contralateral (direct pathway) to the stimuli are subtracted from that in the hemisphere ipsilateral (callosal pathway), providing a measurement of IHTT. This methodology makes it possible to compare IHTT in two directions (i.e., left-to-right hemisphere transfer; right-to-left hemisphere transfer).

Numerous studies of IHTT have shown that neural information travels more quickly from the right hemisphere of the brain to the left hemisphere, than from left hemisphere of the brain to right hemisphere in neurologically healthy adults (e.g., [29,30,31,32]). Miller [33] has proposed that the right hemisphere of the brain contains a greater number of heavily myelinated axons than the left hemisphere, enhancing its performance in fast parallel processes. Studies combining electrophysiology and anatomical imaging have shown that the speed of hemispheric transfer is inversely correlated with fractional anisotropic values in the posterior corpus callosum [34], suggesting that greater callosal integrity may result in quicker hemispheric transfer. In this study we predicted that the asymmetry between left-to-right IHTT and right-to-left IHTT would be reduced, or non-existent in expert VGPs, reflecting a more balanced connectivity between the left and right hemisphere. Consistent with previous literature, non-VGPs were expected to show a quicker right-to-left IHTT than left-to-right IHTT.

Electrophysiological studies also allow for the study of absolute N1 latencies. The absolute latency is the latency of the evoked potential appearing in the hemispheric contralateral to the visual field in which the stimulus was presented. N1 latencies along this direct pathway have been suggested to reflect the time taken to discriminate visual stimuli [35] and lengthen as the attentional burden of an experimental task increases [36]. Previous research on expert VGPs consistently shows that VGPs possess quicker responses to visuospatial stimuli. Due to the link between the absolute N1 latency of the direct visual pathway and the discriminative processing of attended stimuli [34], it is possible that commonly observed quicker behavioural responses shown by expert VGPs may be partially underpinned by more rapid low-level visual processing.

We also predicted that any changes to electrophysiological measures shown by expert VGPs would be the result of extensive video-game play. Consequently we also calculated correlations between electrophysiological measures (IHTT asymmetry; absolute N1 latency) and video-gaming characteristics (age begun; years of experience; hours per week).

Finally we compared the reaction times of expert VGPs and non-VGPs to the visual stimuli. This was primarily to test whether this sample of VGPs showed the usual advantage in speed of responding and reduced effects of hand dominance on response speed.

## Methods

### 2.1) Ethics Statement

Ethics approval for this study was obtained from the University of Auckland Human Participants Ethics Committee. Written informed consent was obtained from all participants prior to testing. All participants were naïve to the study’s hypotheses. The experiment was performed in the Research Center for Cognitive Neurosciences high density EEG facility within the School of Psychology of The University of Auckland.

### 2.2) Participants

Fifteen male expert video-game players and 16 matched non-VGPs participated in the study. Participants were recruited through public advertisement and via advertisement of opportunities to participant in research in the School of Psychology at the University of Auckland. Video-gamers were defined as expert if they began playing before the age of 10, had a minimum 8 years of experience and a minimum play time of 20 hours per week over the last 6 months. Game types currently played by VGPs were restricted to first-person shooters (e.g., *CounterStrike: Global Offensive*), real-time strategy (e.g., *StarCraft II*), action real-time strategy (e.g., *Defense of the Ancients 2*), massively multiplayer online role-playing (e.g., *Guild Wars 2*), and others which utilize the mechanics of the aforementioned game genres but are not aggressive (e.g., the *Portal* series). These game genres require swift bimanual movements in response to complex in-game visual cues and commonly employ advanced artificial intelligence and multiplayer capabilities. The expert VGP group on average began playing at the age of 5.80 (SE = .42), with 17.47 (SE = .97) years of experience and a mean 34.67 hours (SE = 5.01) of play-time per week over the last 6 months. Non-VGPs were required to have little-to-no video-game experience (maximum of 1.5 years). Individuals were excluded if they were left handed. Although females were eligible to participate, no females were recruited who met the criterion to be included as an expert VGP. There were no statistical differences between the expert VGPs and non-VGPs for age (expert VGPs: M = 23.27, SE = .88; non-VGPs: M = 25.69, SE = 1.19; t(29) = 1.62, p = .12), years of education (expert VGPs: M = 16.33, SE = 1.19; non-VGPs: M = 16.53, SE = .71; t(29) = .15, p = .89) or handedness as established by the Edinburgh Handedness Inventory [37] (expert VGPs: M = 93.87, SE = 1.68; non-VGPs: M = 91.35; SE = 2.62, t(25.27) = .81, p = .43). There were also no significant differences between the two groups on measures of estimated verbal IQ (expert VGPs: M = 111.00, SE = 2.12; non-VGPs: M = 113.50, SE = 3.10; t(26) = .67, p = .51), performance IQ (expert VGPs: M = 120.86, SE = 1.52; non-VGPs: M = 118.53, SE = 2.39; t(27) = -.81, p = .43) or estimated full scale IQ (expert VGPs: M = 117.50, SE = 1.79; non-VGPs: M = 118.07, SE = 2.71; t(26) = .18, p = .86), as assessed by the Wechsler Abbreviated Scale of Intelligence (WASI) [38].

### 2.3) Materials and general procedure

Stimuli were black/white checkerboard circles with a diameter of 3° visual angle and presented for 92 ms against a gray background using E-Prime. At the widest diameter of the circle there were 17 checkerboard squares. Stimuli appeared in either the left visual field or right visual field with their midpoint 6° from the central fixation cross.

EEG was recorded continuously at a 1 kHz sampling rate (0.1-400 Hz analogous band pass) using a high density 128-channel Ag/AgCl electrode net (Electrical Geodesics Inc., Eugene, OR, USA). Impedances for all electrode channels were kept below 40 kΩ. Data were acquired using a common reference electrode (Cz), positioned anatomically, and were later re-referenced to the average.

Participants were tested within a quiet, electrically shielded Faraday chamber and were seated 57 cm away from a 22 inch Samsung computer monitor (1920x1080 pixel resolution; 60 Hz refresh rate; 13 ms lag in display) on which stimuli were presented. Throughout the experiment participants were instructed to maintain their gaze on the centrally located fixation cross. An initial block of 12 practice trials was followed by four experimental blocks, with hand order set to: right hand, left hand, left hand, right hand. Stimuli were preceded by a variable interstimulus interval of 542 ms, 742 ms or 942 ms. Following presentation of a stimulus participants responded by pressing the space bar. Each block contained a total of 70 trials which were randomized between 30 presentations to the left visual field, 30 presentations to the right visual field and 10 catch trials (no stimulus). Catch trials were included to ensure participants maintained focus on the task. Participants were provided with an opportunity to rest at the beginning of each block, where they were also instructed on which hand to use next.

### 2.4) Analyses

#### 2.4.1) Behavioural data

Reaction data was collected at a 1 ms resolution. Correct responses were defined as those key presses that occurred after a stimulus presentation. Response errors occurred when participants did not respond to a stimulus presentation or responded to a catch trial. Participant accuracy was calculated as the number of correct responses divided by the total number of stimulus, expressed as a percentage. Analysis of reaction time and accuracy data were evaluated with separate repeated measures ANOVA with group (expert VGPs; non-VGPs) as the between-subjects factor and hand (left hand; right hand), and visual field (left visual field; right visual field) as within-subject factors.

#### 2.4.2) EEG data

**Figure 1:**
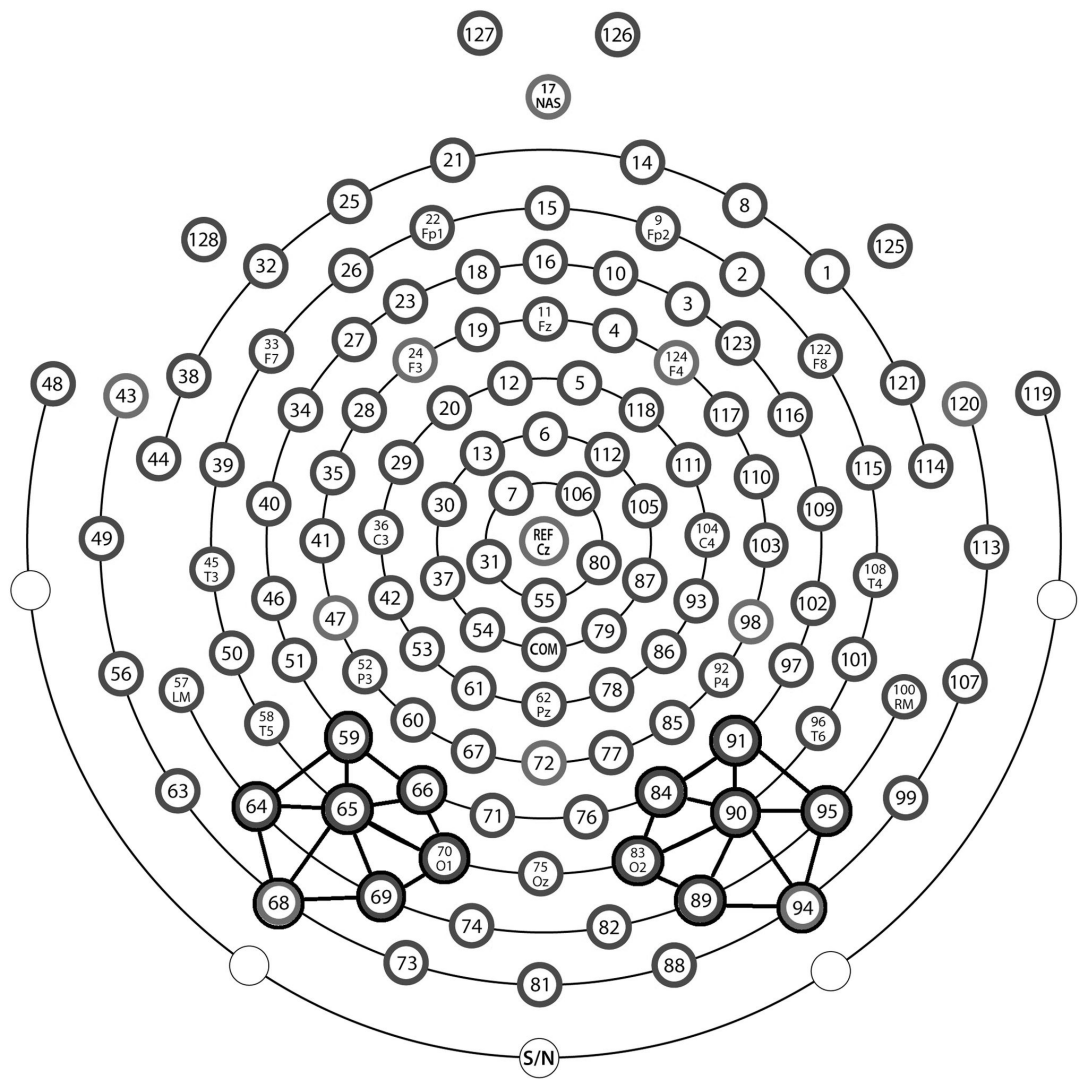
Diagram of Electrical Geodesic 128-electrode net (standard 10-20 system). Black circles and black line connectors show electrode clusters used for left and right hemisphere.

EEG was segmented into epochs 100 ms pre-stimulus onset to 400 ms post-stimulus onset. Electrodes located at the outer canthi and above and below the left and right eyes were used to calculate horizontal and vertical electrooculogram (EOG). Recordings contaminated by vertical eye-movements and eye blinks (vertical EOG amplitudes exceeding ±70 µV), and horizontal eye-movements (horizontal EOG amplitudes exceeding ±70 µV) were discarded from the analysis. The remaining trials were corrected for residual eye movement artifacts using procedures from Jervis et al. (1985) [39]. The mean number of epochs remaining for expert VGPs was 92.47 (SE = 3.54) for the left visual field and 94.60 (SE = 3.65) for the right visual field, and for non-VGPs was 72.38 (SE = 5.83) for the left visual field and 73.69 (SE = 6.05) for the right visual field. Independent samples t-tests revealed the expert VGPs had significantly more epochs for analyses than the non-VGPs for both the left visual field, t(24.51) = -2.95, p = .007, and the right visual field, t(24.45) = -2.96, p = .007. Data were re-filtered to 30 Hz lowpass offline and average evoked potentials were constructed for left visual field and right visual field conditions. The N1 component of the evoked potential was defined as the greatest peak of the first negative wave that occurred at least 140 ms following stimulus presentation. N1 latencies were recorded from each participant using a cluster of seven lateral occipital electrodes (chosen *a priori*) centered between P3, T5 and O1 in the left hemisphere and between P4, T6 and O2 in the right hemisphere (standard 10-20 system) and averaged (see Figure 1). Interhemispheric transfer time estimates were calculated for each individual participant by subtracting the latency of the contralateral N1 (direct) from the latency of the ipsilateral N1 (following callosal transfer) for both left visual field and right visual field conditions.

## Results

### 3.1) Behavioural Analyses

Analysis of reaction time data revealed a significant main effect of group, F(1, 29) = 4.24, p = .049. As expected, expert VGPs responded (M = 274.50, SE = 8.16) significantly more quickly than non-VGPs (M = 297.88, SE = 7.90) to the presentation of lateralized visual stimuli. There was a significant interaction between hand and visual field, F(1,29) = 15.28, p = .001, with responses to stimuli in the right visual field made significantly more quickly with the right hand than the left hand (p = .005), while responses made to stimuli in the left visual field were made significantly faster with the left hand than the right hand (p = .03). Responses made with the right hand were significantly faster to stimuli in the right visual field than the left visual field (p = .01). Contrary to predictions, however, there was no significant difference between reaction times to stimuli presented in the left visual field between the left hand and the right hand (p = .92).

**Figure 2:**
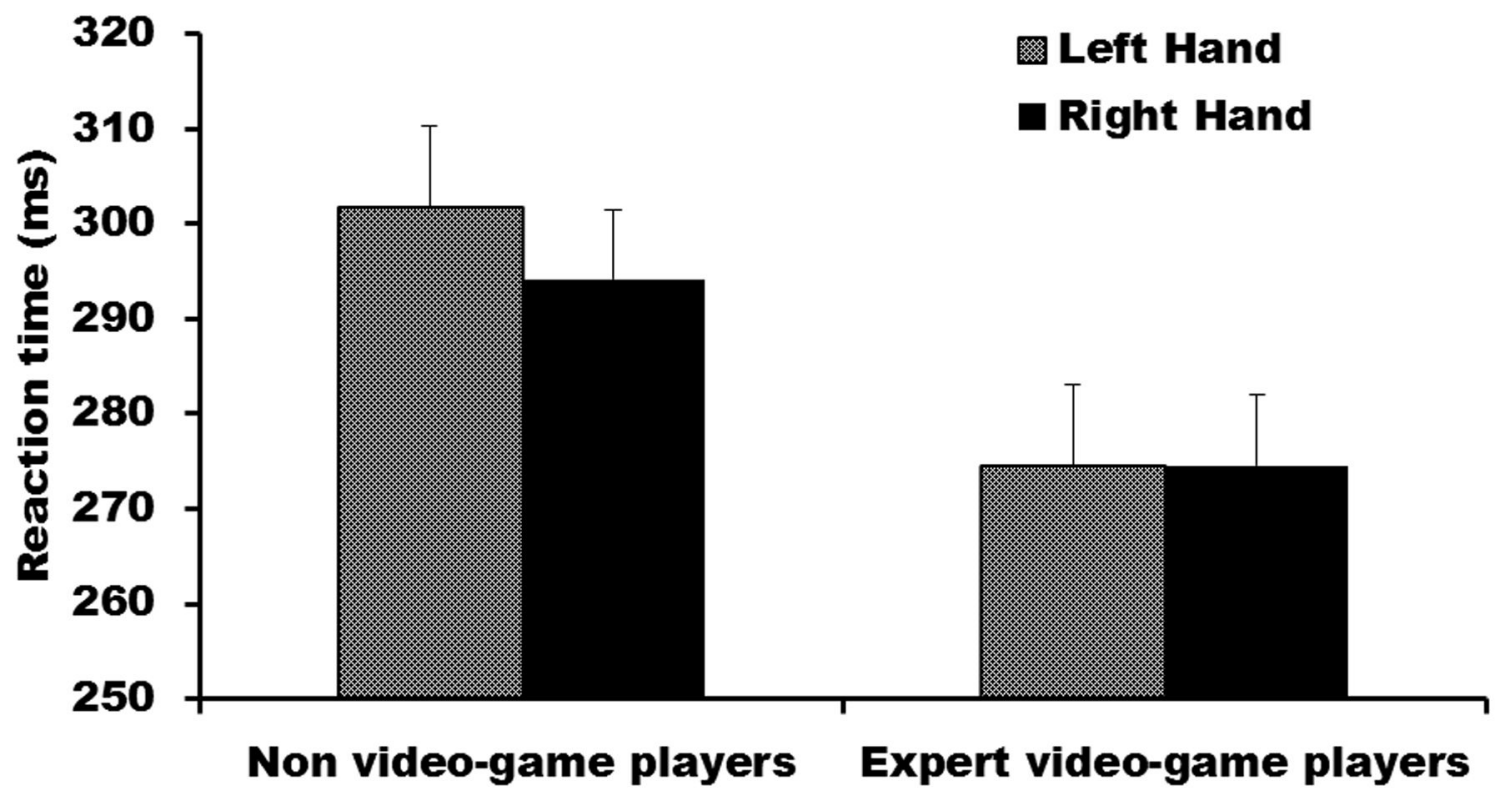
Mean reaction time by hand used for expert video-game players and non-video-game players. Error bars represent standard error.

There were no other significant main effects or significant interactions although the main effect of hand approached significance, F(1,29) = 3.21, p = .08, as did the hand by group interaction, F(1,29) = 3.41, p = .08. Figure 2 shows expert VGPs responded as quickly with their left as their right hand, whereas Non-VGPs tended to respond more quickly with their right hand.

Accuracy data for expert VGPs and non-VGPs were analysed to make sure that expert VGPs and non-VGPs performed the task accurately and comparably. This was confirmed. Accuracy for both expert VGPs (M = 98.22, SE = .28) and non-VGPs (M = 98.98, SE = .27) was high and no significant main effects for group, hand or visual field, or significant interactions were found (all p-values > .05).

### 3.2) EEG

#### 3.2.1) IHTT

**Figure 3:**
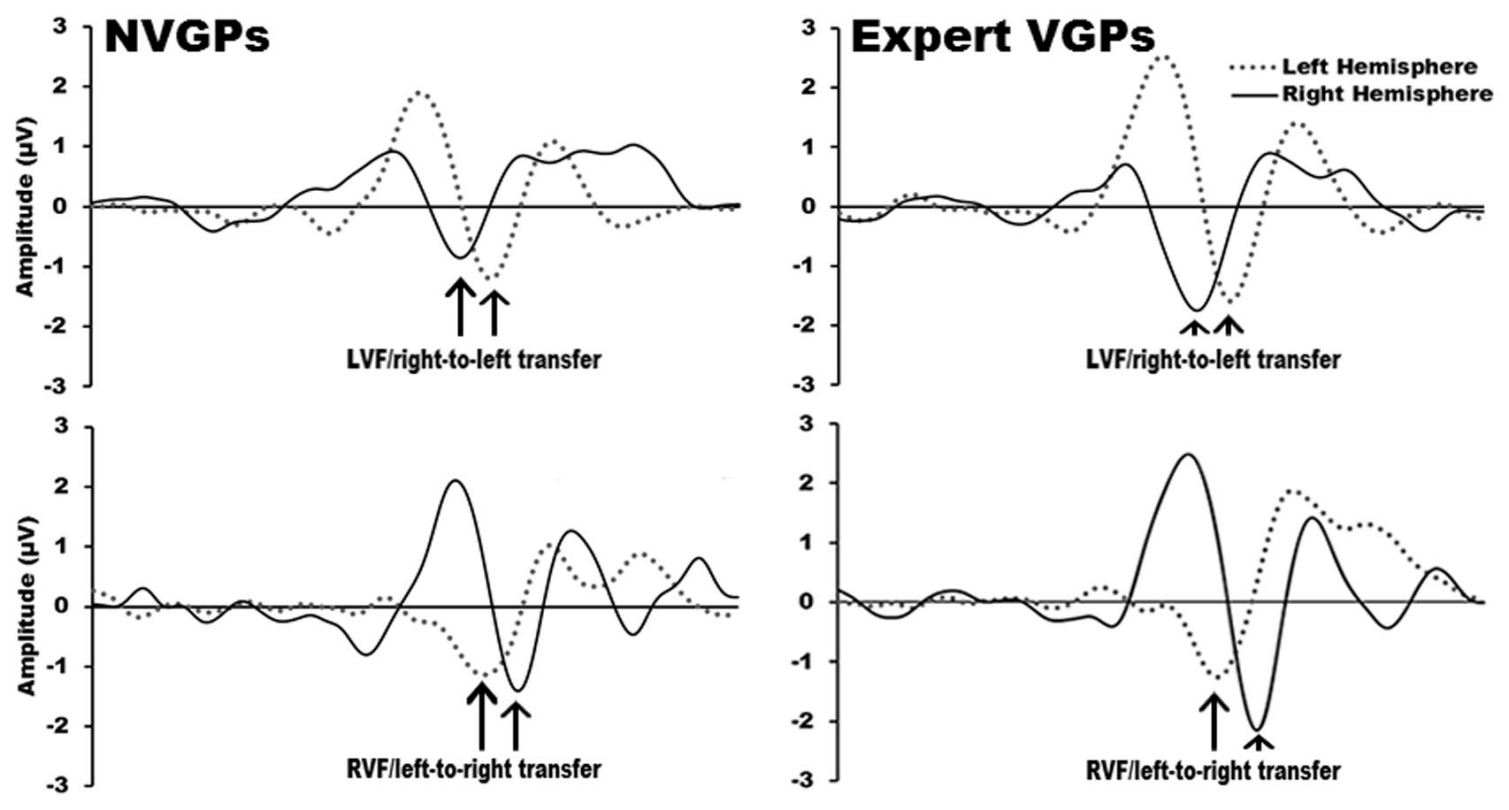
Grand mean waveforms in left and right hemisphere occipital electrode clusters for non-video-game players and expert video-game players during stimulus presentation in the left and right visual field.

Effects for IHTT were analyzed using a repeated measures ANOVA with group (expert VGPs; non-VGPs) as the between-subjects factor and direction (left-to-right transfer; right-to-left transfer) as a within-subjects factor. Grand mean wave forms for contralateral (direct) and ipsilateral (indirect pathway) N1s elicited by left and right visual field stimuli in expert VGPs and non-VGPs are shown in Figure 3.

**Figure 4:**
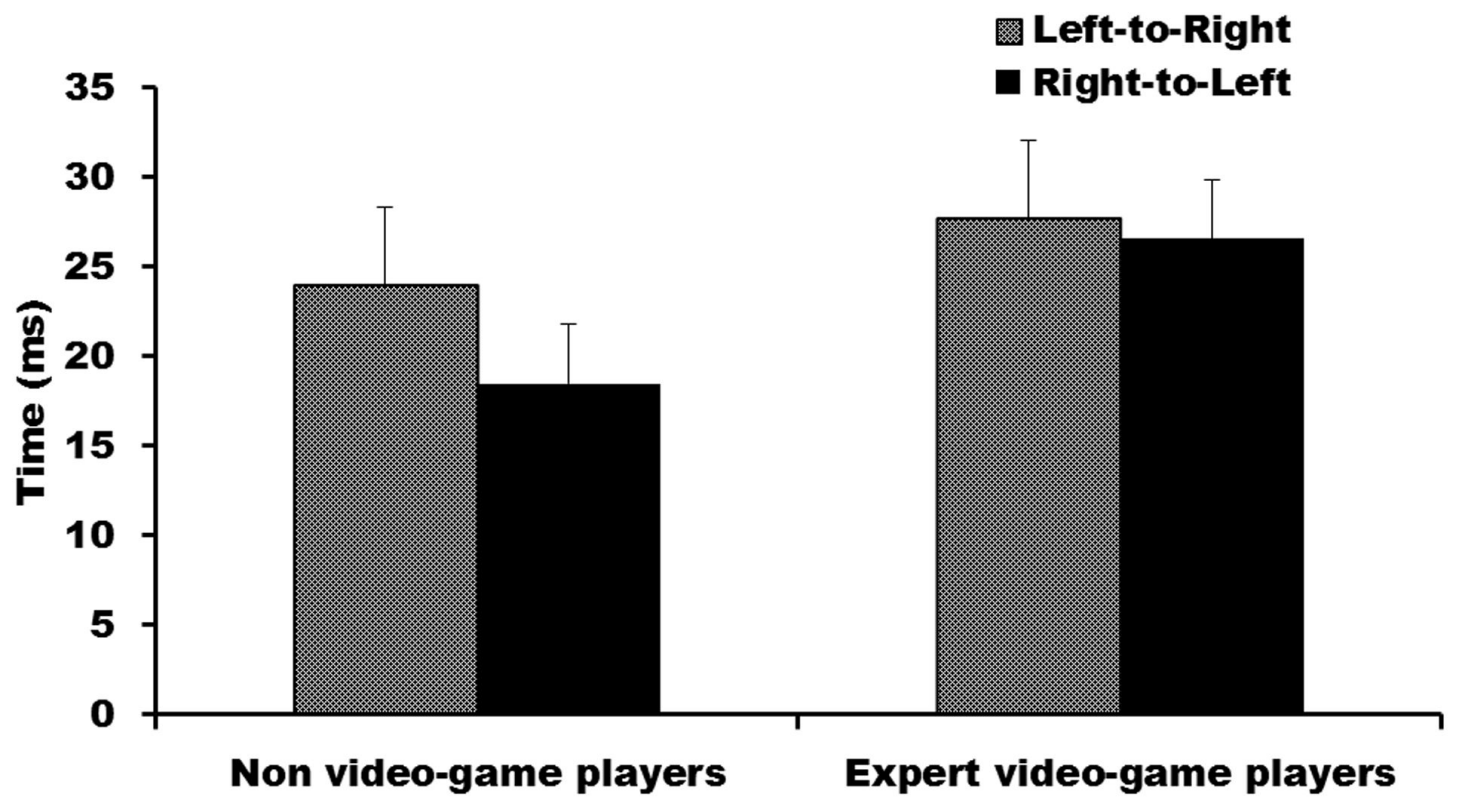
Mean interhemispheric transfer time for each direction for expert video-game players and non-video-game players. Error bars represent standard error.

The ANOVA for IHTT did not reveal a significant main effect of group, F(1,29) = 1.56, p = .22, nor, surprisingly, a significant main effect of transfer direction, F(1,29) = 1.33, p = .26. Furthermore, although non-VGPs appeared to show the expected faster transfer from right-to-left than left-to-right in Figure 4, there was no significant interaction between group and transfer direction, F(1,29) = .57, p = .46. Together this suggests that neither expert VGPs, nor non-VGPs, had faster IHTTs when transferring visual information from the right-to-left hemisphere.

#### 3.2.2) Absolute latency of the N1

**Figure 5:**
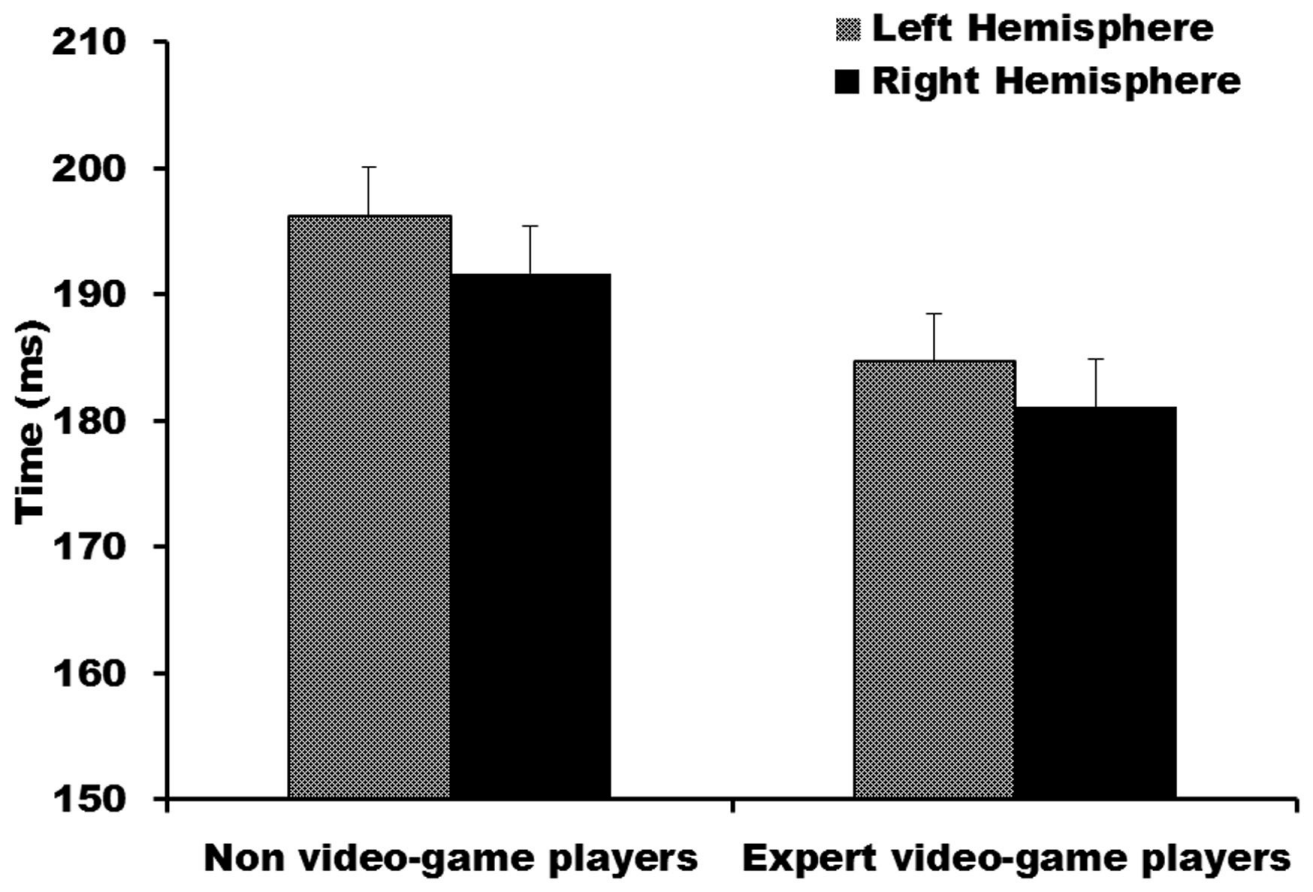
Mean absolute N1 latency for direct pathways for each hemisphere for expert video-game players and non-video-game players. Error bars represent standard error.

N1 latencies for direct pathways only (contralateral visual fields and hemispheres) were analysed with a repeated measures ANOVA with group (expert VGPs; non-VGPs) as the between-subjects factor and hemisphere (left hemisphere; right hemisphere) as a within-subjects factor. The main effect of group was significant, F(1,29) = 4.87, p = .04 (see Figure 5), with expert VGPs displaying significantly earlier N1 latencies (M = 182.92, SE = 3.60) than non-VGPs (M = 193.99, SE = 3.49). No other effects were significant (all p-values > 0.5).

If earlier N1 latencies influence speed of behavioral responding, then these two variables should be positively correlated. In other words as absolute N1 latency increases, so too should behavioural response times. This was confirmed by a significant Pearson’s correlation between the grand average of absolute N1 latency and response time, r = .39, p = .03. Finally, Pearson’s correlation coefficients were calculated within the expert VGP group to assess the relationship between video gaming characteristics (age began regular gaming; years of experience; hours per week) and mean absolute N1 latency. It was predicted that for expert VGPs, as years of experience and number of hours gaming increased, absolute N1 latency should show a relative decrease. Contrary to predictions no Pearson’s correlation was found to be significant.

## Discussion

This study is the first electrophysiological investigation of IHTT, a measure of callosal function, and absolute occipital N1 latencies of expert VGPs. Using the latencies of N1 responses we measured IHTT speeds in both directions for both expert VGPs and non-VGPs. As predicted, expert VGPs showed no directional advantage for IHTT, indicating relatively equilateral transfer of visual information across the corpus callosum in the two directions (right-to-left hemisphere and left-to-right). Contrary to predictions, however, non-VGPs showed a similar degree of symmetry in their IHTTs for transfer of visual information. Notably expert VGPs showed significantly earlier absolute N1 latencies in both hemispheres than non-VGPs.

As expected the behavioural responses of expert VGPs were quicker than those of non-VGPs. Expert VGPs responded equally quickly with both hands, while non-VGPs tended to respond slightly more quickly with their right than left hands. These findings are consistent with previous studies investigating VGPs (e.g., [1,2,3]), and in this sense confirm that this sample of VGPs has behavioural characteristics in-line with those of VGPs in previous research. Modern video-game play requires players to translate complex visual cues into precise and rapid bimanual movements. Performance is continually adjusted based on incoming sensory feedback and occurs under a constant time pressure. Extended game-play often begins during early childhood and continues through much of adolescence, a period in which the brain is developing and at its most malleable. Thus the ability of expert VGPs to make quicker behavioural responses may result from prolonged training. An alternative account, however, is that the quicker responses of expert VGPs are not the result of video-game play, but instead reflect a pre-existing characteristic that allows these individuals to become expert VPGs.

Another behavioural difference between the groups was that expert VGPs had significantly more good EEG epochs available for analyses, although the numbers available for both groups were high. This difference reflects fewer trials were contaminated (and rejected) by eye movements, blinks or facial musical contractions in expert VGPs, suggesting gamers are better at staring impassively at the computer screen, which again may reflect prolonged game-play.

The key finding in the current study is that expert VGPs have earlier N1 latencies than non-VGPs in the direct pathways of both hemispheres. The latency of the N1 component of a visual event related potential has been suggested to reflect the time taken for an individual to discriminate visually-attended stimuli [35] and typically lengthens as the attentional demands of an experimental task increase [36]. Thus shorter N1 latencies may enable expert VGPs to discriminate attended visual stimuli significantly earlier than non-VGPs and contribute to faster responding in visual tasks. Additionally as expert VGPs can successfully perform the same visual task as non-VGPs with a shorter N1 latency, they may be able to make more efficient use of limited attentional resources in the visual domain. Certainly there is existing literature showing enhanced performance by VGPs on tasks involving attentional abilities in the visual domain (e.g., [1,2,3]). The earlier N1 time-course in expert VPGs may underpin some of the temporal enhancements seen in tasks such as attentional blink, temporal order judgment and backwards masking (e.g., [5,6,7]). The significant correlation between N1 latency and response time also suggests shorter N1 latencies may contribute to the faster responses of expert VGPs during visual tasks. One explanation for this electrophysiological enhancement in expert VGPs is that it results from training in the form of sustained video-game play. Successful video-game play requires players to continuously attend to and classify visual stimuli as relevant or irrelevant to in-game goals while under constant time pressure. Constant play while under these conditions, overtime, may facilitate an ability to identify attended visual stimuli earlier and make more efficient use of attentional resources in the visual domain. An alternative account, however, is that individuals who become expert VGPs do so because they can discriminate visual stimuli earlier and use attentional resources in the visual domain more efficiently than their peers, resulting in more successful game-play.

Estimates of IHTT are constructed by subtracting the latency of evoked potentials in the hemisphere contralateral to the visual field in which the stimulus is presented (direct pathway) from the latency of evoked potentials ipsilateral to the presented stimulus (indirect or callosal pathway). This methodology also makes it possible to compare IHTT in two directions (i.e., left-to-right transfer; right-to-left transfer). Previous IHTT literature has frequently shown that neural information transfers faster right-to-left than from left-to-right in healthy adults (e.g., [29,30,31,32]). In the current study we predicted that this typical asymmetry in expert VGPs relative to non-VGPs would be reduced, or non-existent, reflecting more balanced neural connectivity between the left and right hemisphere. Contrary to predictions there was no significant difference in the speed of callosal transfer between expert VGPs and non-VGPs, nor in the relative speed of transfer in the two directions. Although the pattern of transfer speeds in the non-VGP group was in the expected direction of faster transfer from right-to-left, this was not significant; both expert VGPs and non-VGPs showed relatively equilateral speed of transfer of visual information across the corpus callosum. No evidence in the current study exists to suggest that there are any significant group differences between expert VGPs and non-VGPs in callosal function.

The absence of the predicted asymmetry in IHTT times in our non-VGP group was somewhat surprising. However, while a number of studies have shown the expected IHTT asymmetry (quicker right-to-left IHTT than left-to-right IHTT) in adult populations, other patterns of IHTT have also been reported. A number of sub-populations appear to show a reduced or absent asymmetry in IHTT, for example, expert musicians [28], individuals with attention deficit hyperactivity disorder both inattentive and combined subtypes [40], females [41] and left-handers [42]. Additionally, Whitford and colleagues [43] failed to find an IHTT asymmetry in their healthy controls using N1 latencies derived from current source density headmaps. Surprisingly, a reversed IHTT asymmetry (i.e., quicker left-to-right IHTT than right-to-left IHTT) was found when calculated using P1 latencies. The non-VGPs in this study provide another example of absent asymmetry in IHTT in a healthy adult sample.

We failed to find any significant correlations between video-gaming characteristics and absolute N1 latencies within the expert VGP group. This may reflect the low variability within our expert VGP sample in both the age at which they began gaming, years of gaming and hours per week spent gaming. If the criteria for inclusion in the VGP sample was expanded to include VGPs who started to play after the age of 10 and played casually for less than 20 hours per week there may have been a higher likelihood of significant correlations. Many video-game training studies have shown that even a small period of video-game play can result in behavioural enhancements in the same direction as those seen in expert VGPs (e.g., [1,44,45,46,47]) and these enhancements last for at least 5 months [11,12].

In conclusion, this study provides evidence, for the first time, that expert gamers have faster neural processing of visual stimuli than non-VGPs. This provides the basis for future training studies designed to test whether or not sustained training can shorten occipital N1 latencies, and whether this facilitates the ability to identify attended visual stimuli earlier, enabling faster responding during visual tasks.
